# Continuous labor support by doulas and nurses and its impact on birth outcomes: a systematic review

**DOI:** 10.3389/frhs.2026.1889587

**Published:** 2026-09-23

**Authors:** Mirfat Mohamed Labib Elkashif, Doaa Mostafa Sheashaa, Magda Mahmoud Mohammad Elgameel, Mohamed Sayed Abdellatif, Ahmed Abd-Elrahman Mohamed, Sally Abd-Elrahman Mohamed

**Affiliations:** 1 Department of Nursing Sciences, College of Applied Medical Sciences in Wadi Aldawaser 18616, Prince Sattam Bin Abdulaziz University, Alkharj, Saudi Arabia; 2 Lecturer of Obstetric and Gynecological Nursing, Faculty of Nursing, Delta University for Science & Technology, Gamasa, Egypt; 3 Department of Psychology, College of Education in Al-Kharj, Prince Sattam Bin Abdulaziz University, Alkharj, Saudi Arabia; 4 Pediatric Resident, Mansoura International Hospital, Dakhlia, Egypt

**Keywords:** continuous labor support, doula, maternal outcomes, neonatal outcomes, nurse and midwife support

## Abstract

**Background:**

Continuous labor support provided by doulas, nurses, or midwives is increasingly recognized as an important component of respectful, woman-centered maternity care. Evidence suggests that continuous support during labor may improve maternal and neonatal outcomes by enhancing emotional support, facilitating labor progress, and reducing unnecessary obstetric interventions. However, variations in intervention models and healthcare settings have resulted in inconsistent findings regarding the effectiveness of different support providers. This systematic review aimed to evaluate the impact of continuous labor support provided by doulas, nurses, or midwives on maternal and neonatal outcomes and to identify current evidence gaps to inform clinical practice and future research.

**Methods:**

This systematic review was conducted according to the Preferred Reporting Items for Systematic Reviews and Meta-Analyses (PRISMA 2020) guidelines. Electronic searches were performed in PubMed, CINAHL, and Web of Science to identify eligible studies published between January 2008 and December 2025. Only peer-reviewed primary empirical studies published in English were included. Eligible studies evaluated continuous labor support provided by doulas, nurses, midwives, or trained birth companions and reported maternal and/or neonatal outcomes. Sixteen studies met the inclusion criteria. Data were synthesized narratively according to study characteristics, intervention type, and reported outcomes.

**Results:**

Overall, continuous labor support was associated with improved maternal and selected neonatal outcomes across diverse healthcare settings. The most consistent maternal benefits included increased spontaneous vaginal birth, reduced cesarean delivery, shorter labor duration, lower use of obstetric interventions, improved maternal satisfaction, and enhanced breastfeeding initiation. Midwife-led continuous support demonstrated favorable effects on labor progression, whereas community-based doula programs showed particular benefits for socially disadvantaged and migrant women by improving access to culturally responsive maternity care. Evidence regarding nurse-led continuous labor support remained limited. Although neonatal outcomes generally showed positive trends, findings for Apgar scores and neonatal intensive care unit admission were less consistent across studies.

**Conclusions:**

Continuous labor support appears to be an effective, woman-centered strategy for improving childbirth outcomes and maternal experiences. Integrating doulas, nurses, and midwives into models of continuous labor support may contribute to reducing unnecessary obstetric interventions and promoting respectful maternity care. However, heterogeneity among intervention models and outcome measures limits direct comparison between studies. Future research should prioritize high-quality comparative trials evaluating different support providers, particularly nurse-led models, and investigate long-term maternal and neonatal outcomes.

## 1 Introduction

Improving maternal and neonatal outcomes remains a global public health priority despite substantial advances in obstetric care. Although improvements in medical technologies and evidence-based clinical practices have contributed to reductions in maternal and neonatal morbidity and mortality, increasing rates of obstetric interventions—particularly cesarean birth—have raised concerns regarding the quality and appropriateness of intrapartum care. Many women continue to report negative childbirth experiences characterized by inadequate emotional support, anxiety, poor communication, and fragmented care during labor. Consequently, there has been increasing emphasis on woman-centered maternity care that integrates both high-quality clinical management and continuous supportive care throughout childbirth (1, 2).

Continuous labor support refers to the uninterrupted presence of a trained support person who provides emotional, physical, informational, and advocacy support throughout labor and birth. Such support may be provided by healthcare professionals, including nurses and midwives, or by trained non-clinical companions such as doulas. While nurses and midwives provide both clinical and supportive care, doulas focus primarily on continuous emotional, physical, and informational support without performing clinical procedures. The World Health Organization (WHO) recommends that every woman should have access to a companion of choice during labor as part of respectful maternity care, recognizing continuous support as an essential component of high-quality intrapartum care (2).

Recent evidence continues to support the benefits of continuous labor support across a range of maternal and neonatal outcomes. A comprehensive systematic review and meta-analysis published in 2024, including 35 randomized controlled trials, reported that continuous labor support was associated with higher rates of spontaneous vaginal birth, lower cesarean section rates, shorter labor duration, improved Apgar scores, increased breastfeeding success, and greater maternal satisfaction. The review also demonstrated beneficial psychological effects, including reduced fear of childbirth and improved childbirth experiences, although the magnitude of these effects varied across studies (3). Likewise, recent evidence syntheses have suggested that doula-supported care may contribute to improved maternal experiences through continuous emotional support, advocacy, and facilitation of communication between women and healthcare professionals (4).

The effectiveness of continuous labor support may vary according to the type of support provider. Doulas are specifically trained to provide uninterrupted non-clinical support throughout labor, whereas nurses and midwives combine supportive care with continuous clinical assessment, monitoring, and implementation of obstetric interventions. However, heavy workloads, staffing shortages, and competing clinical responsibilities may limit nurses' and midwives' ability to provide continuous one-to-one support during labor. Understanding the similarities and differences between these support models is therefore essential for optimizing maternity care services and improving women's childbirth experiences (5).

Despite increasing recognition of its benefits, continuous labor support remains inconsistently implemented across healthcare systems. Organizational barriers, limited workforce capacity, financial constraints, institutional policies, and highly medicalized models of childbirth continue to limit women's access to continuous supportive care. These challenges are particularly evident in low- and middle-income countries, where shortages of skilled maternity care providers and high patient volumes reduce opportunities for individualized labor support (2, 5).

Although several systematic reviews have examined continuous labor support, important knowledge gaps remain. Many previous reviews have focused primarily on doula care or evaluated companionship during labor without adequately distinguishing between support provided by doulas, nurses, and midwives. In addition, considerable heterogeneity exists regarding intervention characteristics, provider type, healthcare settings, and reported maternal and neonatal outcomes. Consequently, an updated systematic review focusing on recent primary evidence is needed to better understand the effectiveness of different continuous labor support models and their impact on birth outcomes.

Therefore, this systematic review aims to synthesize and critically evaluate the current evidence regarding the role of continuous labor support provided by doulas and nurses/midwives on maternal and neonatal birth outcomes. By examining different support models and their effects on clinical and psychosocial outcomes, this review seeks to inform evidence-based maternity practice, healthcare policy, and future research while supporting the implementation of high-quality, respectful, and woman-centered maternity care.

## 2 Methods

### 2.1 Aim

This systematic review aimed to synthesize and critically evaluate evidence from primary studies on the effectiveness of continuous labor support provided by doulas, nurses, midwives, or other trained companions in improving maternal and neonatal outcomes during labor and childbirth.

Specifically, the review sought to:

(a) Identify and describe models of continuous labor support (e.g., doula-led, nurse-led, midwife-led, or trained companion support) implemented during intrapartum care across different healthcare settings.
(b) Evaluate the effects of continuous labor support on maternal outcomes, including mode of birth (spontaneous vaginal birth, cesarean birth, and instrumental birth), duration of labor, labor pain, use of pharmacological analgesia, obstetric interventions, and maternal satisfaction with the childbirth experience.
(c) Assess the impact of continuous labor support on neonatal outcomes, including Apgar scores, neonatal intensive care unit (NICU) admission, breastfeeding initiation, and immediate neonatal well-being.
(d) Compare the reported maternal and neonatal outcomes associated with different types of labor support providers, particularly doulas and nurses/midwives, where evidence was available.
(e) Identify trends, methodological limitations, and research gaps regarding the implementation, timing, and key components of continuous labor support interventions.
(f) Provide evidence-based recommendations for clinical practice, maternity care policy, and future research to facilitate the integration of effective continuous labor support into routine intrapartum care.

### 2.2 Design

This systematic review was conducted in accordance with the **Preferred Reporting Items for Systematic Reviews and Meta-Analyses (PRISMA) 2020 Statement** to ensure transparent, rigorous, and reproducible reporting of the review process. The review followed a predefined methodology for study identification, screening, eligibility assessment, data extraction, and evidence synthesis.

The review focused exclusively on **primary empirical studies** evaluating continuous labor support provided by doulas, nurses, midwives, or other trained birth companions and its effects on maternal and neonatal outcomes during labor and childbirth. Secondary evidence, including systematic reviews, meta-analyses, scoping reviews, narrative reviews, and opinion articles, was excluded to avoid duplication of evidence and ensure that only original research findings were synthesized.

### 2.3 Eligibility criteria

The eligibility criteria were established according to the **Population, Intervention, Comparison, Outcomes, and Study Design (PICOS)** framework.

#### 2.3.1 Inclusion criteria

Studies were included if they met all of the following criteria:

- Included pregnant women during labor and childbirth.
- Evaluated continuous labor support provided by doulas, nurses, midwives, or other trained birth companions.
- Reported at least one maternal or neonatal outcome related to continuous labor support.
- Used a primary empirical study design, including randomized controlled trials, quasi-experimental studies, prospective or retrospective cohort studies, case-control studies, or qualitative studies.
- Published in peer-reviewed journals.
- Published in English.
- Published between **January 2008 and December 2025**.

#### 2.3.2 Exclusion criteria

Studies were excluded if they:

- Were systematic reviews, meta-analyses, scoping reviews, umbrella reviews, narrative reviews, editorials, conference abstracts, commentaries, protocols, dissertations, or letters.
- Focused exclusively on antenatal or postpartum interventions without evaluating labor support during childbirth.
- Did not investigate continuous labor support provided by doulas, nurses, midwives, or trained birth companions.
- Did not report maternal or neonatal birth outcomes.
- Were not published in English.
- Were published before January 2008 or after December 2025.

### 2.4 Information sources and search strategy

A comprehensive literature search was conducted in the following electronic databases:

- PubMed
- CINAHL (via EBSCOhost)
- Web of Science Core Collection

The search covered studies published from **January 2008 through December 2025**. The final database search was completed in **March 2026**.

The search strategy combined Medical Subject Headings (MeSH) and free-text keywords using Boolean operators (AND, OR). The principal search terms included:

(“continuous labor support” OR “continuous labour support” OR “labor companionship” OR “birth companion” OR doula* OR “doula support” OR “nurse support” OR “midwife support”)

AND

(“labor” OR childbirth OR birth)

AND

(“maternal outcomes” OR “birth outcomes” OR “neonatal outcomes” OR “mode of birth” OR “cesarean section” OR breastfeeding OR “Apgar score”)

Database-specific adaptations of the search strategy were applied where appropriate.

The retrieved citations were exported into **EndNote 21 (Clarivate Analytics)** for reference management and duplicate identification prior to screening.

### 2.5 Study selection and screening

Study selection followed the PRISMA 2020 recommendations.

After duplicate records were removed, two reviewers independently screened the titles and abstracts of all retrieved studies according to the predefined eligibility criteria.

Potentially eligible studies were subsequently retrieved in full text and independently assessed by the same reviewers.

Disagreements regarding study eligibility were resolved through discussion and consensus. When consensus could not be reached, a third reviewer was consulted to make the final decision.

The study selection process was documented using a PRISMA 2020 flow diagram that summarized the numbers of identified, screened, excluded, and included studies.

## 3 Results

### 3.1 Characteristics of the included studies

A total of **16 primary studies** met the eligibility criteria and were included in this systematic review (Figure 1). The included studies were published between **2008 and 2025** and consisted of **nine randomized controlled trials (RCTs), five cohort studies, one comparative clinical study, and one prospective observational study**.

**Figure 1:**
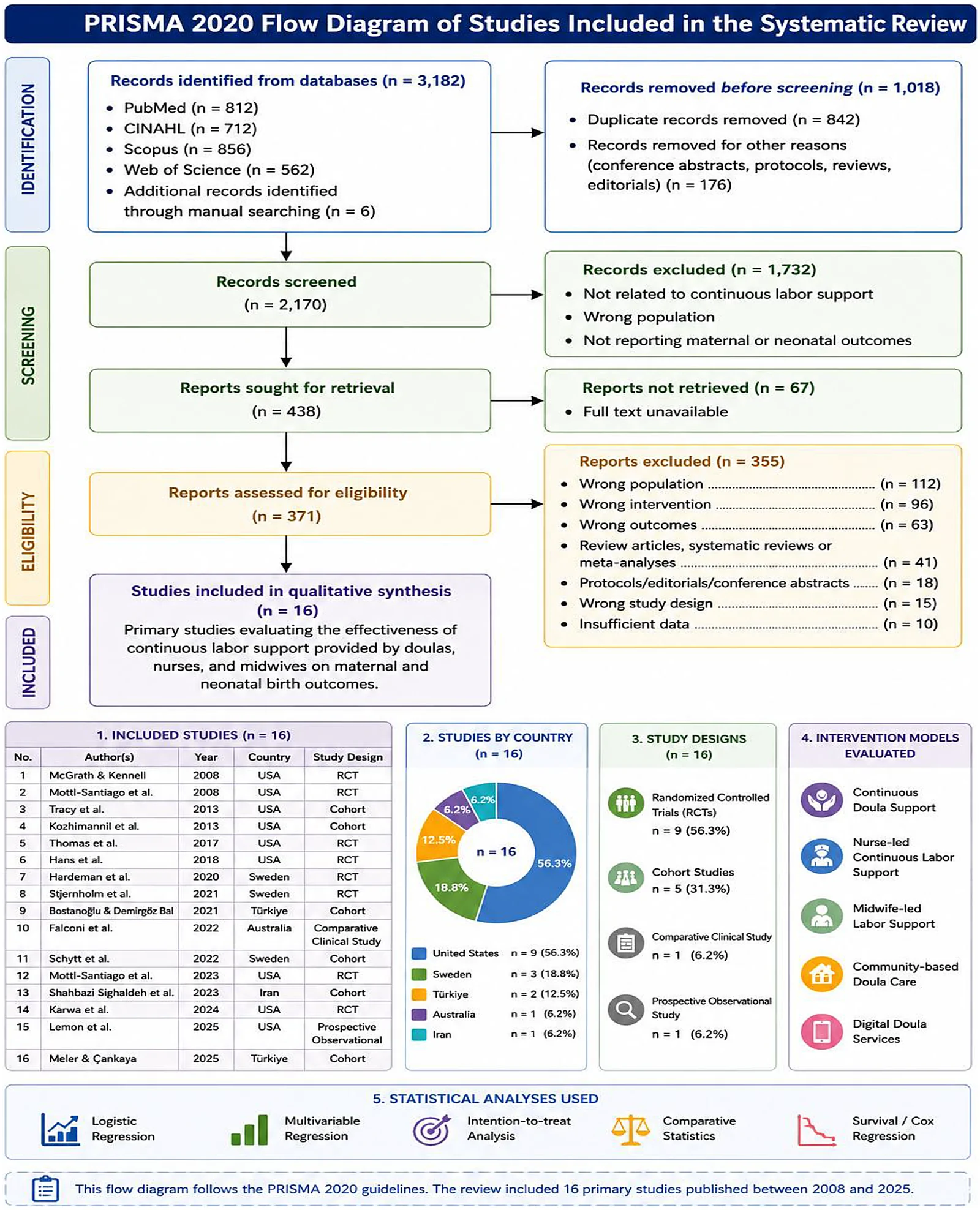
PRISMA flow diagram of studies included for review.

Most studies were conducted in the **United States (*n*** **=** **9)**, followed by **Sweden (*n*** **=** **3)**, **Türkiye (*n*** **=** **2)**, **Australia (*n*** **=** **1)**, and **Iran (*n*** **=** **1)**. Continuous labor support was provided by **doulas, nurses, midwives, or trained birth companions** in hospital- and community-based maternity care settings. The interventions evaluated included hospital-based doula programs, community-based doula care, continuous midwife-led labor support, nurse-led labor support, and digital doula services.

The primary outcomes assessed across the included studies were mode of birth, duration of labor, obstetric interventions, maternal satisfaction, breastfeeding outcomes, neonatal outcomes, maternal psychological well-being, and health system outcomes. Detailed characteristics of the included studies are presented in Table 1.

**Table 1:** Characteristics of the included studies on continuous labor support (*n* = 16).

Country	Author	Year	Purpose	Study design	Sampling/setting	Intervention	Statistical analysis	Key finding
United States	Hans, S. L., Edwards, R. C., & Zhang, Y.	2018	To evaluate the effectiveness of an innovative doula-home-visiting intervention on birth outcomes, postpartum maternal health, infant health, and newborn care practices among young, low-income mothers.	Randomized Controlled Trial (RCT)	Young pregnant women (mean age = 18.4 years) from four Illinois communities in the United States. Participants were randomly assigned to receive either doula-home-visiting services (*n* = 156) or case-management services (*n* = 156).	The intervention integrated community doulas into an evidence-based home-visiting program. Doulas provided prenatal home visits, childbirth education, breastfeeding counseling, continuous labor support, and postpartum home visits up to 6 weeks after birth.	Intention-to-treat analysis using logistic regression models. Odds ratios (ORs) with 95% confidence intervals (CIs) were calculated to compare maternal and infant outcomes between groups.	Women receiving doula-home-visiting services were significantly more likely to attend childbirth education classes (50% vs. 10%; OR = 9.82), less likely to use epidural or pharmacologic pain medication during labor (72% vs. 83%; OR = 0.49), and more likely to initiate breastfeeding (81% vs. 74%; OR = 1.72). They also demonstrated improved newborn care practices, including safe sleep positioning and appropriate car-seat use. No significant differences were observed in cesarean delivery, preterm birth, birthweight, or postpartum depression.
United States	Hardeman, R. R., et al.	2020	To evaluate the effectiveness of community-based doula care in improving maternal and birth outcomes among socially disadvantaged women.	Community-based cohort study	Pregnant women receiving community-based doula services in the United States.	Continuous community-based doula support during pregnancy, labor, and postpartum.	Logistic regression and multivariable regression analyses.	Community-based doula care was associated with improved maternal outcomes, reduced birth inequities, and increased access to respectful maternity care.
Sweden	Stjernholm, Y. V., Charvalho, P. S., Bergdahl, O., Vladic, T., & Petersson, M.	2021	To investigate whether continuous labor support provided by a midwife improves labor progress and increases vaginal delivery among primiparous women.	Randomized Controlled Trial	59 primiparous women.	Continuous one-to-one labor support by a midwife.	Regression analysis, Student's *t*-test.	↓ Active labor duration; ↑ vaginal birth; ↓ maternal cortisol; ↑ maternal satisfaction.
Türkiye	Bostanoğlu, G., & Demirgöz Bal, M.	2021	To evaluate the effects of continuous labor support provided by a midwife on maternal anxiety, labor duration, and childbirth outcomes.	Randomized Controlled Trial (RCT)	70 primiparous women randomized to continuous midwife support (*n* = 35) or routine care (*n* = 35).	Continuous labor support by a midwife throughout labor.	Chi-square test, independent *t*-test, and Mann–Whitney U test.	↓ Anxiety; ↑ coping with labor pain; ↓ labor duration by approximately 2 h; ↓ labor interventions and perineal trauma.
United States (California, etc.)	Falconi, A. M., et al.	2022	To evaluate the association between doula care across the maternity care continuum and maternal health outcomes using Medicaid claims data.	Retrospective cohort study with propensity score matching	Medicaid claims data from 2014 to 2020. A matched cohort of **298 women receiving doula care** and **298 controls** from three U.S. states.	Continuous doula care during pregnancy, labor, birth, and the postpartum period through community doula programs.	Propensity score matching, logistic regression, odds ratios (ORs), and 95% confidence intervals (CIs).	Doula care was associated with **52.9% lower odds of cesarean birth** (OR = 0.47; 95% CI: 0.29–0.79) and **57.5% lower odds of postpartum anxiety/depression** (OR = 0.43; 95% CI: 0.22–0.82).
Sweden	Schytt, E., Wahlberg, A., Eltayb, A., Tsekhmestruk, N., et al.	2022	To evaluate the effectiveness of community-based bilingual doula (CBD) support on migrant women's intrapartum care experiences and postnatal emotional well-being.	Randomized Controlled Trial (RCT)	164 pregnant migrant women recruited from six antenatal clinics and five hospitals in Stockholm, Sweden.	Community-based bilingual doula support during labor and birth in addition to standard maternity care.	Intention-to-treat analysis, logistic regression, odds ratios (ORs) with 95% confidence intervals (CIs), and comparison of means.	No significant improvement in overall childbirth experience or postnatal emotional well-being compared with standard care; the intervention was feasible and acceptable for migrant women.
United States	Mottl-Santiago, J., Dukhovny, D., Cabral, H., Rodrigues, D., Spencer, L., Valle, E. A., & Feinberg, E.	2023	To evaluate whether an enhanced community doula intervention improved maternal and neonatal outcomes among publicly insured pregnant women receiving care in a safety-net hospital.	Pragmatic Randomized Controlled Trial	Nulliparous, low-risk pregnant women receiving publicly funded insurance at Boston Medical Center, USA. Participants were randomized to an enhanced Birth Sisters community doula intervention or routine maternity care.	Enhanced community doula care beginning at approximately 24 weeks' gestation, including prenatal home visits, continuous labor support, postpartum home visits, health education, and navigation of health and social services.	Intention-to-treat analysis using logistic regression to estimate odds ratios (ORs) with 95% confidence intervals; subgroup analyses by race/ethnicity were also performed.	No statistically significant overall reduction in cesarean birth was observed. However, among Black non-Hispanic participants there was a 12% absolute reduction in cesarean birth and an 11.5% increase in exclusive breastfeeding during the birth hospitalization. A trend toward higher breastfeeding initiation was also observed.
Iran	Shahbazi Sighaldeh, S., Azadpour, A., Vakilian, K., Rahimi Foroushani, A., Vasegh Rahimparvar, S. F., & Hantoushzadeh, S.	2023	To compare maternal outcomes among women receiving care from professional doulas, trained lay companions, and routine midwifery care during labor.	Quasi-experimental comparative study	150 women with low-risk singleton pregnancies who planned vaginal birth. Participants were allocated to three equal groups (doula, trained lay companion, routine midwifery care) at public hospitals and private clinics in Arak, Iran.	Participants were allocated to three groups: doula care, trained lay companion, or routine midwifery care during labor and childbirth.	Chi-square test, Fisher's exact test, One-way ANOVA, and Kruskal–Wallis test.	Doula care significantly reduced labor duration and improved maternal satisfaction. Trained lay companion support significantly reduced maternal anxiety. No significant differences were observed in labor pain among the three groups.
United States	Karwa, S., Jahnke, H., Brinson, A., Shah, N., Guille, C., & Henrich, N.	2024	To examine the association between virtual doula care and birth outcomes among users of a comprehensive digital health platform.	Retrospective cohort study	8,989 pregnant individuals enrolled in a U.S. digital health platform between January 2020 and April 2023.	Virtual doula care delivered through a digital health platform during pregnancy.	Multivariable logistic regression, Chi-square test, Fisher's exact test, and *t*-test.	Women completing ≥2 virtual doula visits had lower odds of cesarean birth, greater emotional support, improved birth decision-making, and better pregnancy education than non-users.
United States	Lemon, L. S., Quinn, B., Young, M., Keith, H., Ruscetti, A., & Simhan, H. N.	2025	To quantify the association between doula care and maternal and neonatal outcomes across different obstetric populations.	Retrospective cohort study	Women delivering at a tertiary academic medical center in the United States (January 2021–December 2022).	Continuous doula care during pregnancy, labor, and birth.	Multivariable logistic regression and subgroup analyses.	Doula care was associated with lower cesarean birth and preterm birth rates, improved maternal outcomes, and favorable neonatal outcomes, particularly among high-risk women.
Türkiye	Meler, K. M., & Çankaya, S.	2025	To evaluate the effect of a WHO-based intrapartum care model delivered by midwives on labor outcomes and women's childbirth experiences.	Randomized Controlled Trial (RCT)	Low-risk pregnant women receiving hospital-based maternity care in Türkiye.	WHO-recommended evidence-based intrapartum supportive care provided by midwives.	Comparative statistical analyses with significance testing between intervention and control groups.	↓ Labor pain; ↓ fear of childbirth; ↑ labor comfort; ↓ oxytocin use; ↓ labor duration; ↑ positive perception of midwifery care.

### 3.2 Maternal outcomes

#### 3.2.1 Mode of birth

Seven studies evaluated the effect of continuous labor support on mode of birth. Most studies reported lower cesarean birth rates among women receiving continuous labor support than among those receiving routine maternity care (6–12). Several studies also demonstrated higher rates of spontaneous vaginal birth, particularly when continuous one-to-one support was maintained throughout active labor (6, 9, 11, 13–15).

Although the overall direction of evidence favored continuous labor support, the magnitude of benefit varied across intervention models and healthcare settings.

#### 3.2.2 Duration of labor

Four studies examined labor duration (6, 9, 14, 15). Most reported significantly shorter active labor among women receiving continuous labor support compared with routine care. Midwife-led continuous labor support appeared particularly effective in facilitating normal labor progression and reducing labor duration.

#### 3.2.3 Obstetric interventions

Six studies evaluated obstetric interventions during childbirth (6–9, 15, 16). Overall, continuous labor support was associated with reduced use of epidural analgesia, oxytocin augmentation, and instrumental birth. However, the magnitude of these reductions varied across studies, and some reported no statistically significant differences.

#### 3.2.4 Maternal satisfaction and childbirth experience

Eight studies assessed maternal satisfaction and women's childbirth experiences (10, 11, 15, 17–21). Women receiving continuous labor support generally reported greater emotional support, improved communication with healthcare providers, increased participation in decision-making, and higher overall satisfaction than women receiving standard maternity care.

Community-based doula programs also improved women's perceptions of respectful and culturally appropriate maternity care, particularly among underserved and migrant populations (19–21).

### 3.3 Neonatal outcomes

Eight studies evaluated neonatal outcomes (8–12, 15, 20, 21). Improved breastfeeding initiation and continuation were the most consistently reported neonatal benefits (8, 10, 12).

Several studies also reported higher Apgar scores and improved neonatal adaptation following continuous labor support (9, 15, 16). Evidence regarding neonatal intensive care unit (NICU) admission and neonatal morbidity was less consistent, with some studies reporting reductions while others found no statistically significant differences (11, 20, 21).

### 3.4 Maternal psychological outcomes

Six studies investigated maternal psychological outcomes (10, 15, 18–21). Overall, women receiving continuous labor support experienced lower anxiety levels, reduced fear of childbirth, greater confidence during labor, and more positive childbirth experiences than women receiving routine care.

Several studies also reported improvements in maternal self-efficacy and early mother–infant bonding following birth (10, 19, 21). Nevertheless, psychological outcomes were assessed using different measurement tools, limiting direct comparisons between studies.

### 3.5 Health system outcomes

Five studies examined health system outcomes (7, 11, 12, 19, 20). Continuous labor support was associated with lower healthcare utilization through reductions in cesarean birth, fewer obstetric interventions, and shorter hospital stays.

Community-based doula programs also demonstrated the potential to improve equitable access to maternity care for socially disadvantaged populations (19, 20). However, several studies highlighted implementation challenges, including workforce shortages, limited reimbursement policies, and inadequate integration of doulas within routine maternity services (11, 20).

### 3.6 Comparison of continuous labor support models

The included studies evaluated several distinct models of continuous labor support that differed in provider type, care setting, and scope of support. Community-based doula programs primarily targeted underserved and migrant women and emphasized culturally responsive care; some programs also incorporated prenatal education, care navigation, and postpartum support in addition to intrapartum care (10, 19–21). Hospital-based doula services primarily focused on continuous emotional, informational, and physical support during labor and birth (8, 17). Midwife-led models represented a clinical form of continuous support and demonstrated favorable effects on labor progression, spontaneous vaginal birth, and maternal satisfaction (9, 13–15). Where explicitly reported, some midwife-led interventions provided continuous one-to-one support throughout labor; however, staffing ratios or whether midwives were exclusively assigned to a single woman were not consistently reported and were therefore not inferred. One included study directly compared professional doula care, trained lay-companion support, and routine midwifery care, highlighting differences between these models of labor companionship (16). In addition, one study evaluated virtual doula support delivered through a digital health platform, representing a model that differs from continuous physical bedside support. Overall, these findings indicate that continuous labor support is not a uniform intervention but encompasses different provider roles, care settings, and models of delivery.

### 3.7 Approach to risk of bias assessment

Risk of bias was assessed descriptively across the included primary studies based on key methodological domains, including study design, adequacy and clarity of the study sample, description of the continuous labor support intervention, appropriateness and clarity of outcome measurement, and the presence of a comparison or control group. Greater methodological rigor was considered to be present in studies using randomized or comparative designs, clearly defined interventions, adequately described samples, appropriate outcome measures, and clearly reported comparison groups.

The included randomized and comparative studies generally demonstrated stronger methodological characteristics, particularly when interventions were clearly defined and maternal and neonatal outcomes were systematically assessed. However, several observational, qualitative, and descriptive studies had methodological limitations, including smaller or insufficiently reported samples, reliance on self-reported outcomes, lack of control or comparison groups, variability in intervention implementation, and limited follow-up. These limitations may increase the potential for selection bias, reporting bias, and residual confounding and should therefore be considered when interpreting the findings.

Overall, methodological heterogeneity was evident across the included primary studies. This variability, together with differences in study design, intervention characteristics, outcome measures, and healthcare settings, limited the ability to make definitive comparisons across studies and contributed to the decision not to conduct a quantitative meta-analysis. Nevertheless, the consistency of findings across several study designs and settings provides supportive evidence for the potential benefits of continuous labor support while highlighting the need for more rigorously designed comparative studies.

### 3.8 Overall summary of findings

Overall, the included studies suggest that continuous labor support provided by doulas, nurses, or midwives is associated with improvements in several maternal and neonatal outcomes. The most consistently reported benefits included increased maternal satisfaction, higher rates of spontaneous vaginal birth, shorter labor duration, reduced obstetric interventions, improved breastfeeding outcomes, and enhanced maternal psychological well-being (6–21).

Nevertheless, variations in intervention characteristics, study populations, healthcare settings, and outcome measures contributed to heterogeneity across the included studies. Therefore, while the overall evidence supports the integration of continuous labor support into routine maternity care, the magnitude of its effects should be interpreted with appropriate caution.

## 4 Discussion

This systematic review synthesized evidence from 16 primary studies published between 2008 and 2025 to evaluate the effects of continuous labor support provided by doulas, nurses, midwives, or trained birth companions on maternal and neonatal outcomes. Overall, the findings suggest that continuous labor support is associated with improved maternal experiences and favourable clinical outcomes, although the magnitude of benefit varied according to the type of support provider, intervention model, and healthcare setting.

One of the most consistent findings across the included studies was the association between continuous labor support and improved mode of birth. Women who received continuous one-to-one support were generally more likely to achieve spontaneous vaginal birth and less likely to undergo caesarean delivery. These findings are biologically and clinically plausible because continuous emotional reassurance, physical comfort measures, and effective communication may reduce maternal stress, facilitate labor progression, and decrease unnecessary obstetric interventions. Similar conclusions have been reported in recent systematic reviews and meta-analyses demonstrating that continuous labor companionship contributes to lower intervention rates and improved childbirth outcomes (1, 3).

The present review also found that continuous labor support was associated with shorter labor duration and reduced use of medical interventions, including epidural analgesia and oxytocin augmentation. **This difference may be related to the broader clinical role of midwives as maternity care providers, which enables them to influence aspects of labor management and the care environment that may facilitate physiological birth. In contrast, doula continuous support was associated with greater maternal confidence and other social-emotional outcomes during childbirth.** These findings support current recommendations advocating continuous one-to-one support throughout labor as an essential component of respectful maternity care (2).

Maternal satisfaction emerged as another consistently improved outcome. Women receiving continuous support reported feeling more respected, informed, and involved in decision-making during childbirth. Continuous presence by doulas or midwives appeared to strengthen communication between women and healthcare professionals while promoting a greater sense of control over the childbirth experience. These findings are consistent with recent qualitative evidence indicating that emotional support and advocacy are central mechanisms through which continuous labor support improves women's childbirth experiences (5).

The review also demonstrated favorable neonatal outcomes associated with continuous labor support. Improved breastfeeding initiation was the most consistent neonatal finding, while several studies also reported higher Apgar scores and better early neonatal adaptation. These benefits are likely to result indirectly from improved maternal well-being, reduced obstetric interventions, and enhanced early mother–infant interaction rather than from the labor support itself. Nevertheless, evidence regarding neonatal morbidity and NICU admission remained inconsistent, suggesting that these outcomes may be influenced by multiple clinical factors beyond labor companionship.

An important contribution of this review is the recognition that continuous labor support should not be considered a uniform intervention, as the role and professional scope of the support provider differed substantially across the included studies. Doulas and trained birth companions primarily provided non-clinical emotional, informational, physical, and advocacy support, whereas nurses and midwives provided supportive care within broader clinical maternity-care responsibilities. Consequently, the mechanisms through which support may influence childbirth experiences and outcomes may differ according to the provider's professional role.

Community-based doula programs appeared particularly relevant for socially disadvantaged, migrant, and underserved women by providing culturally responsive support and reducing barriers to maternity care. In contrast, midwife-led models combined continuous supportive care with clinical maternity expertise and demonstrated favorable effects on physiological labor outcomes, including labor duration and spontaneous vaginal birth. The direct comparison of professional doula support with trained lay-companion and routine midwifery care also highlights that different forms of non-clinical companionship should not be assumed to be equivalent. Evidence regarding nurse-led continuous labor support remained comparatively limited, highlighting an important gap in the current literature.

An important finding of this review was the limited amount of contemporary primary research directly comparing different models of continuous labor support. Despite the longstanding evidence supporting continuous labor companionship, only 16 primary studies met the eligibility criteria over the period covered by this review, and relatively few directly compared different types of support providers or models of care. This evidence gap is important because continuous labor support is implemented within diverse maternity-care systems, with substantial differences in provider roles, staffing structures, professional scope, and organization of care. The limited number of comparative studies may therefore reflect the complexity of evaluating and comparing support models across different healthcare settings. Future research should move beyond establishing whether continuous support is beneficial and should more systematically examine which models, provider characteristics, and organizational conditions are most effective for different populations and maternity-care contexts.

The findings should be interpreted considering several methodological considerations. Considerable heterogeneity existed among the included studies regarding intervention components, duration of support, provider qualifications, outcome measures, and healthcare settings. Such variability limited direct comparison between studies and precluded quantitative pooling of results. Furthermore, although randomized controlled trials comprised the largest proportion of included studies, several observational studies were also included, introducing potential risks of selection bias and residual confounding.

### 4.1 Implications for practice

The findings support integrating continuous labor support into routine maternity services as part of woman-centered and respectful maternity care. Healthcare organizations should consider expanding access to appropriately trained doulas and birth companions while also promoting organizational models that enable nurses and midwives to provide continuous one-to-one support whenever feasible, recognizing the distinct clinical and non-clinical roles and professional scopes of these providers.

### 4.2 Strengths and limitations

This review has several strengths. It included only primary empirical studies, thereby avoiding duplication of evidence from previous systematic reviews and addressing an important methodological concern. In addition, the review incorporated multiple study designs and included evidence from different countries and healthcare systems, enhancing the breadth of available evidence.

However, several limitations should be acknowledged. The included studies demonstrated substantial heterogeneity in intervention models, study populations, and outcome measures. Only English-language publications were included, which may have introduced language bias. Moreover, relatively few studies specifically evaluated nurse-led continuous labor support, and direct head-to-head comparisons between different provider models were limited. This restricted our ability to determine whether differences in outcomes were attributable to the provider's professional role, the specific support model, or the characteristics of the care setting.

## 5 Conclusion

Continuous labor support provided by doulas, nurses, or midwives appears to improve several maternal and neonatal outcomes, particularly maternal satisfaction, spontaneous vaginal birth, labor progression, and breastfeeding initiation. Community-based doula programs also show promise for improving equity in maternity care among vulnerable populations. However, variations in intervention models and healthcare contexts indicate that further high-quality comparative studies are needed, particularly those evaluating nurse-led continuous labor support and long-term maternal and neonatal outcomes. Future research should also investigate the implementation of sustainable models of continuous labor support within routine maternity services across diverse healthcare systems.
